# Photoacoustic-based approach to surgical guidance performed with and without a da Vinci robot

**DOI:** 10.1117/1.JBO.22.12.121606

**Published:** 2017-08-24

**Authors:** Neeraj Gandhi, Margaret Allard, Sungmin Kim, Peter Kazanzides, Muyinatu A. Lediju Bell

**Affiliations:** a University of Virginia, Department of Electrical and Computer Engineering, Charlottesville, Virginia, United States; b Smith College, Department of Physics, Northampton, Massachusetts, United States; c Johns Hopkins University, Department of Computer Science, Maryland, United States; d Johns Hopkins University, Department of Electrical and Computer Engineering, Baltimore, Maryland, United States; e Johns Hopkins University, Department of Biomedical Engineering, Baltimore, Maryland, United States

**Keywords:** minimally invasive surgery, robotic surgery, photoacoustic image guidance, surgical navigation

## Abstract

Death and paralysis are significant risks of modern surgeries, caused by injury to blood vessels and nerves hidden by bone and other tissue. We propose an approach to surgical guidance that relies on photoacoustic (PA) imaging to determine the separation between these critical anatomical features and to assess the extent of safety zones during surgical procedures. Images were acquired as an optical fiber was swept across vessel-mimicking targets, in the absence and presence of teleoperation with a research da Vinci Surgical System. Vessel separation distances were measured directly from PA images. Vessel positions were additionally recorded based on the fiber position (calculated from the da Vinci robot kinematics) that corresponded to an observed PA signal, and these recordings were used to indirectly measure vessel separation distances. Amplitude- and coherence-based beamforming were used to estimate vessel separations, resulting in 0.52- to 0.56-mm mean absolute errors, 0.66- to 0.71-mm root-mean-square errors, and 65% to 68% more accuracy compared to fiber position measurements obtained through the da Vinci robot kinematics. Similar accuracy was achieved in the presence of up to 4.5-mm-thick *ex vivo* tissue. Results indicate that PA image-based measurements of the separation among anatomical landmarks could be a viable method for real-time path planning in multiple interventional PA applications.

## 1 Introduction

Surgeries typically incur the risk of death and paralysis due to injury to unseen blood vessels and nerves, respectively. Two examples of surgeries that suffer from the lack of real-time visualization of these critical features include mastoidectomies and endonasal transsphenoidal surgeries. In mastoidectomies, surgeons operate near the facial nerve with potential nerve damage leading to facial paralysis. As many as 79% of facial nerve ruptures caused by surgery are undiagnosed.^1^ Endonasal transsphenoidal surgery similarly carries the risk of injury if surgeons rupture either branch of the internal carotid artery while drilling through the sphenoid bone.^2^ Surgery to correct this arterial injury has 14% morbidity and 24% to 40% mortality rates.^3^^,^^4^

These minimally invasive surgeries utilize intraoperative endoscopic camera images to visualize superficial anatomical structures and preoperative imaging modalities, such as CT and MRI, for guidance and navigation around major subsurface vessels.^5^^,^^6^ However, the subsurface vessels might not always match the preoperative images as anatomy may shift during surgery. While intraoperative CT imaging is an option,^7^ it adds risk due to irradiation, adds cost, and has poor resolution. Intraoperative MRI is also possible^8^ and would not incur the radiation risks of CT, but the spatial resolution is similarly poor and the technique is more expensive. Ultrasound (US) is another option for image guidance and visualization, but it requires two-way acoustic propagation and is, therefore, subject to the effects of sound scattering, attenuation, and acoustic clutter during signal transmission and reception. In addition, there is a trade-off between the high-frequency sound waves needed for high-resolution US images and the low frequencies needed for deep acoustic penetration. A similar resolution trade-off exists when comparing the small acoustic aperture sizes that would be excellent candidates for interventional applications with the larger aperture sizes that provide better spatial resolution. Additional challenges with US include poor sensitivity to small vessels and vessels with slow flow (particularly with Doppler US), as well as low sensitivity to nerves.

The field of biophotonics presents new opportunities to guide surgeries using light.^9^^,^^10^ While most of these opportunities are limited by poor optical penetration depths beyond the ballistic region of ∼1  mm, photoacoustic (PA) imaging is particularly promising because light that is successfully transmitted to and absorbed by the surgical site causes acoustic responses that can travel from deeper (i.e., several centimeters) within the body. PA imaging is performed by transmitting pulsed laser light that is absorbed by a target of interest, causing thermal expansion and the subsequent generation of sound waves that are detected with US transducers (also known as US probes).^11^^–^^13^ When the laser is tuned to specific optical wavelengths, blood vessels and nerves will preferentially absorb more of the transmitted light relative to their surroundings, resulting in PA images that contain high-contrast representations of blood vessel and nerve locations.^14^^,^^15^ Note that the peak optical absorption for blood vessels and nerves differ, making it possible to independently visualize one or the other in a single image, although more work is required to demonstrate that this distinction is possible *in vivo*.

When compared to more traditional PA approaches that tend to integrate the light delivery with the US probe, PA imaging is generally more advantageous as a surgical guidance tool when the optical delivery systems are separated from the acoustic receivers, as previously demonstrated for potential PA-guided applications in brachytherapy,^16^^–^^18^ biopsy,^19^ pain management,^15^ fetal surgery,^20^ and neurosurgery.^21^ PA images may additionally be overlaid or interleaved with conventional US images to provide some structural and anatomical context for PA images.

With regard to PA-guided neurosurgery, our previous work shows that PA imaging can be used to visualize blood vessels through bone^21^ by surrounding surgical tools with optical fibers^22^ and utilizing a standard transcranial US probe to receive the acoustic response. With this type of interventional imaging system, the alignment of the separated optical and acoustic hardware (i.e., optical fibers and US probes, respectively) can potentially be controlled with a dedicated navigation system.^23^ Robotic surgery is another possibility to improve minimally invasive surgical outcomes with reduced hand tremors, minimal blood loss, and less time, yet these surgeries similarly suffer from inadequate views of subsurface anatomy. We previously introduced the possibility of augmenting robotic surgery with the benefits of PA imaging using synthetic PA images to illustrate this concept with a research da Vinci Surgical System.^24^ We built on this approach to demonstrate a fully integrated PA and da Vinci system with the goal of having the surgeon use the da Vinci master console as in current procedures, with added information from simultaneous visualization of the endoscopic video and the real-time PA images.^25^

The work presented in this paper is an expanded version of our associated conference paper,^26^ which was the first to investigate the ability of PA imaging to accurately quantify the separation among cylindrical targets, such as blood vessels and nerves, and thereby provide the surgeon with information about safe zones for operation. This approach can be implemented with or without a robot-held surgical tool or a dedicated navigation system. When a robot or navigation system is absent, guidance could be provided by attaching one or more optical fibers to the surgical tool and visualizing surgical tool tips simultaneously with critical anatomical features.^22^ With a robot present (e.g., a teleoperated robot that controls a surgical tool and attached fiber), the robot-based tool tip coordinates can be obtained whenever a critical landmark appears in the PA image. Both methods can provide separation information in the image coordinate system (e.g., separation relative to at least one visualized blood vessel in the PA image), which is the focus of this paper. Although providing similar information in the robot coordinate system is another option when the robot is present,^25^ we avoid relying on this additional information in this paper due to the additional error introduced after performing the required probe calibration^23^ or the required calibration of the fiber with respect to the robot instrument.^25^

There are four primary contributions of this paper. First, we evaluate the accuracy of our approach based on separation measurements obtained from both robotic and nonrobotic image-based measurements. Second, we evaluate the accuracy obtained with both manual and teleoperated control of an optical fiber. Third, we provide a detailed quantitative analysis of discrepancies observed when varying the laser, beamforming method, and fiber control method. Finally, we provide qualitative analyses of variations observed when altering the relative vessel and US probe orientation and when the laser light passes through tissues that range from 1.5 to 4.5 mm in thickness to visualize underlying structures.

## 2 Methods and Materials

### 2.1 Photoacoustic Imaging System

Our PA imaging system consisted of an Alpinion (Seoul, Korea) ECUBE 12R US system attached to an Alpinion L3-8 linear transducer (3- to 8-MHz bandwidth) and one of three lasers that were used in two sets of experiments. The first set of experiments used a portable Laser Components (Olching, Germany) LS Series pulsed laser diode (PLD) and a larger Quantel (Les Ulis, France) Ultra100 Nd:YAG laser. The second set of experiments used the 1064-nm output port from a Phocus Mobile laser configured by Opotek (Carlsbad, California). For all lasers, the laser beam was directed through a 1-mm core diameter, 0.5-numerical aperture (NA) multimode optical fiber after coupling with optical lenses. PA images were displayed in real time during the experiments, and the raw channel data were acquired for postprocessing.

**Fig. 1:**
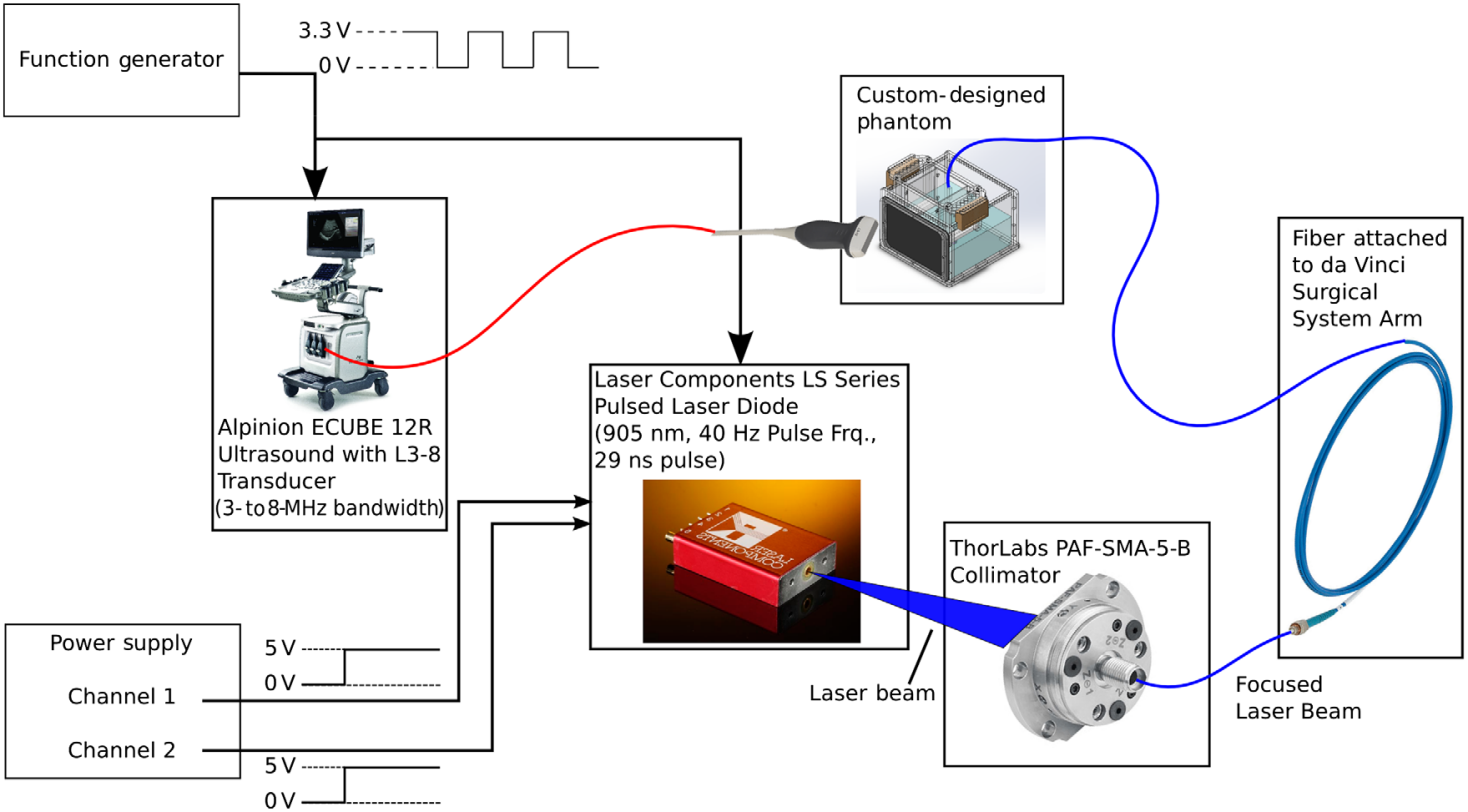
This configuration of our PA imaging system uses the PLD. The optical fiber was both handheld for manual operation and teleoperated when attached to one of the da Vinci surgical tools. A similar setup replaced the PLD with an Nd:YAG laser or Phocus Mobile laser.

The portable 905-nm wavelength PLD was coupled to a collimator connected to a 1-mm core diameter, 0.5-NA optical fiber. The free end of the fiber was both attached to the tip of a teleoperated large needle driver da Vinci tool for teleoperation and detached for handheld (i.e., manual) control of the fiber. A function generator sent a trigger signal to both the US system and the pulsed laser diode at a frequency of 40 Hz. Two channels from a voltage supply were connected to the laser. One 5-V channel controlled the pulse length, while the second 5-V channel controlled the peak power, resulting in a 29-ns pulse length and 226-W peak power, respectively. Thus, the pulse energy of the beam from the laser module was 6.6  μJ. Figure 1 shows the portable laser system synchronized with the US system.

The 1064-nm wavelength laser beam from the Ultra100 Nd:YAG laser was focused through an aspheric lens coupled to the 1-mm core diameter, 0.5-NA optical fiber, which was manually operated and not attached to the da Vinci tool. The laser pulse repetition frequency was 20 Hz with a pulse length of 7 ns. The laser provided an output 5-V transistor–transistor logic (TTL) signal with each pulse firing, which was routed through a function generator and replicated with 3.3-V logic to use as a trigger signal for the acquisition of PA data by the US system. The output laser beam from the optical fiber had a pulse energy of 0.75 mJ.

For the Phocus Mobile laser, the 1064-nm wavelength laser output port was directly coupled to the 1-mm core diameter, 0.5-NA optical fiber using a custom adapter. The fiber was manually operated and not attached to the da Vinci tool. The laser pulse repetition frequency was 10 Hz with a pulse length of 5 ns. The laser provided an output 5-V TTL signal with each pulse firing, which was routed through a function generator and replicated with 3.3-V logic to use as a trigger signal for the acquisition of PA data by the US system. The output laser beam from the optical fiber had a pulse energy of 0.75 to 1.58 mJ.

Note that although we use three different lasers at wavelengths of 905 and 1064 nm, with energies that ranged from 6.6  μJ to 1.58 mJ, our primary purpose is to use these three variations of PA systems to demonstrate the feasibility of a new approach to estimate the distances between critical anatomical landmarks. We assume that this approach can be applied at any wavelength, as long as the vessel or other structures of interest can be seen at the applied wavelength. We also demonstrate the versatility of our method by using these multiple configurations of a PA imaging system.

### 2.2 Photoacoustic System Integration with a da Vinci Robot

**Fig. 2:**
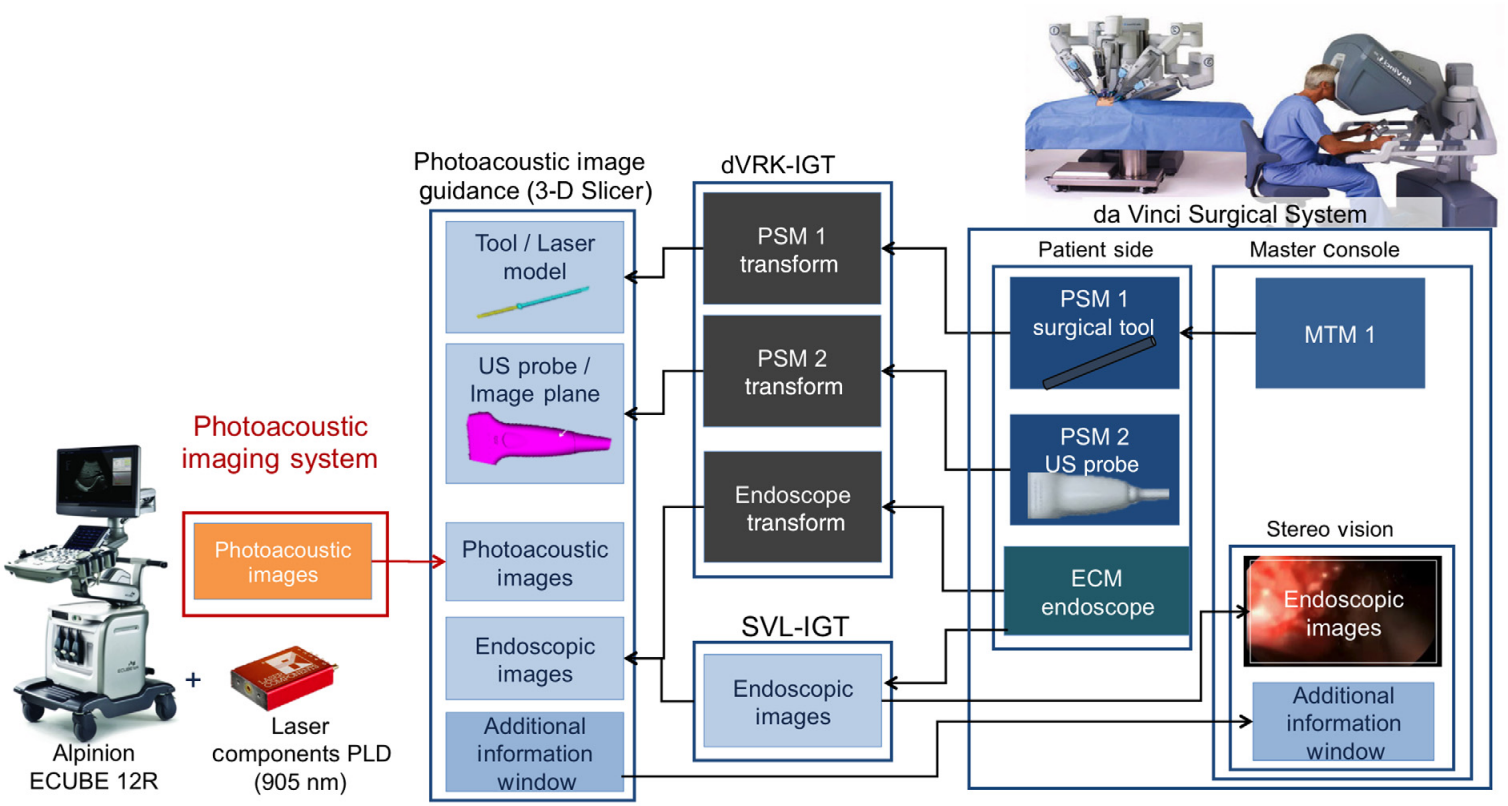
Integrated system architecture. PA images are generated and sent to the PA image guidance module (via a 3-D slicer plug-in) for visualization, along with live stereo endoscope video (via SVL-IGT) and models of the drill, laser, and US probe that are positioned based on kinematic position feedback from da Vinci PSMs (via dVRK-IGT). Visualizations from 3-D Slicer are sent to the da Vinci stereo viewer. Note SVL = cisst Stereo Vision Library^27^ and dVRK = da Vinci Research Kit.^28^

Figure 2 shows how our PA imaging system (left) was interfaced with the da Vinci surgical system (right) for teleoperation of imaging system components, facilitated by the da Vinci research toolkit.^28^ The surgeon sits at the master console, and his or her motions at the console are mimicked by the robotic arms, also known as the patient side manipulators (PSMs). These PSMs can hold various surgical tools. We propose to integrate PA imaging by attaching fibers to the tool and attaching an US probe to one of the PSMs. The manipulator that has the surgical tool and attached fiber can be controlled by a master tool manipulator (MTM). The endoscopic camera manipulator (ECM) controls the endoscope. There are several external software modules that are used to provide data to three-dimensional (3-D) Slicer software via the OpenIGTLink network protocol.^29^ These include an interface to the da Vinci MTM and PSM (i.e., dVRK-IGT) and a stereo endoscope image capture system (i.e., SVL-IGT).

**Fig. 3:**
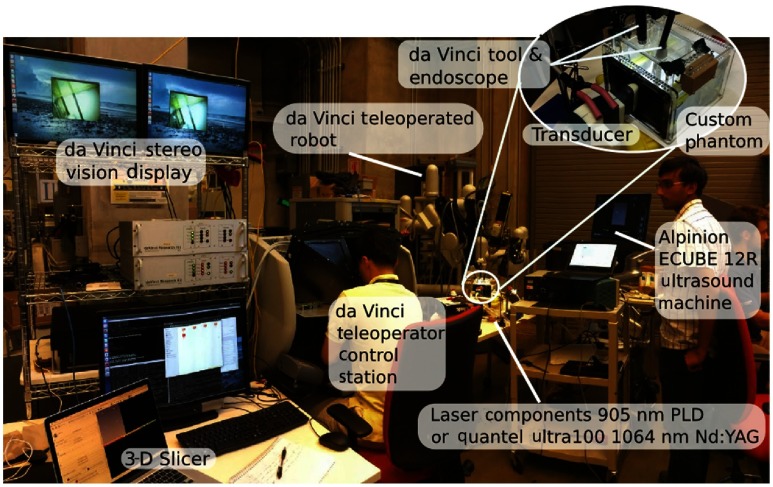
Physical setup when interfacing our PA imaging system with the da Vinci Surgical System. The inset image shows the custom-built phantom. The US transducer was oriented orthogonal to the vessels for this experiment.

Figure 3 shows an image of the experimental setup when integrating our PA imaging system with the da Vinci Surgical System. PSMs are visible near the center of Fig. 3. The system is teleoperated from the master console. Videos from the da Vinci endoscopic camera were transferred to the master console and to the two monitors shown in the upper left corner of Fig. 3. PA images were visible in real time on the screen of the US machine, but images can also be integrated with the master console as we previously proposed^24^ and demonstrated.^25^ The teleoperator used both the endoscopic video and the real-time PA video to position the tool tip.

### 2.3 Custom Modular Phantoms

#### 2.3.1 Best-case scenario

**Fig. 4:**
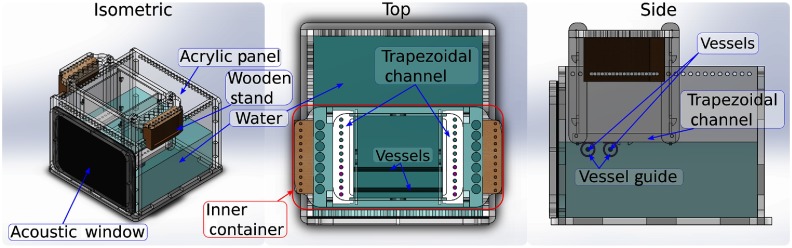
Overview of the phantom design, which consisted of acrylic panels, a rubber acoustic window for US signal reception, and plastic guides to control the vessel separation distances. The two black lines inside the phantom (top view) show a sample arrangement of parallel blood vessels. The phantom was filled with water for PA imaging. The US probe (not shown) was oriented orthogonal to the vessels for this experiment.

To test the image guidance system, we designed and built a custom phantom from laser cut acrylic, 3-D printed components, and wooden stands for height adjustment of the inner container relative to the outer container. Guides for placement of blood vessels were positioned and secured via set screws and enabled modular placement of the vessels during experiments. Figure 4 shows our phantom design with modularity in vessel separation provided by the movable guides shown in Fig. 4 (side). This design builds on the phantom used in previous work, which consisted of inner and outer containers and was used to visualize *ex vivo* bovine blood in the presence of an *ex vivo* sheep brain and *ex vivo* human cadaver skull specimens.^30^ Although these *ex vivo* tissues may be added to the current design, they were not included in the first set of experiments in order to determine accuracy based on the best-case imaging scenario. Tissue is, however, included in the second set of experiments described in Sec. 2.3.2.

The phantom was sealed with silicone and filled with water to permit acoustic wave propagation. The US transducer was fixed against the acoustic window. A black, 3-mm-diameter flexible rubber rod was used to mimic blood vessels. Two parallel blood vessels were placed in the phantom at four separation distances. The vessel separation was measured with calipers each time the vessel configuration was altered. This separation distance ranged from 10.06 to 20.14 mm for the three experiments, which is within the range of expected vessel separations during endonasal transsphenoidal surgery.^31^ The US transducer was fixed in place relative to the acoustic window of the phantom. The optical fiber was pointed at the vessels and could either be submerged in the water or maintained above the water line, as the bottom of the inner container was open to the water.

The upper right corner of Fig. 3 shows a close-up of the custom phantom as used for experiments with the da Vinci robot. The US transducer was fixed to the acoustic window in the orientation shown (i.e., the lateral dimension of the probe was orthogonal to the vessels) using a passive arm. Alternatively, a custom mount can allow one of the da Vinci arms to support the transducer.^25^

#### 2.3.2 Exploring variations in vessel geometry, size, orientation, and tissue thickness

**Fig. 5:**
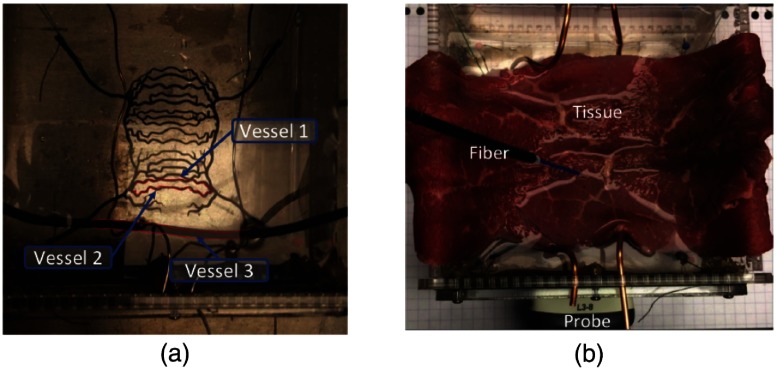
(a) Experimental setup to image a rigid 3-D printed vascular network combined with the flexible vessel from the previous set of experiments. (b) Experimental setup with tissue added to the vascular network phantom. The US probe was oriented both orthogonal (not shown) and parallel to the vessel axes for these experiments.

To investigate more complicated vessel structures, the inner container of the phantom was removed, and a custom-designed 3-D printed model of the arteries around the uterus was suspended by string through the holes at the top of the phantom, as shown in Fig. 5(a), where the three vessels that we imaged are outlined in red (vessels 1 to 3). The vessels in this model are curvy, nonparallel, lie in multiple planes [though this detail is not readily apparent from Fig. 5(a)], and have diameters that range from 1.0 to 1.5 mm, as specified by the 3-D model design. Specifically, vessels 1 and 2 have diameters of 1 mm. Vessel 3 was the same 3-mm-diameter flexible rubber rod used in Sec. 2.3.1, and it was draped across the container to be roughly in the same plane as vessels 1 and 2.

To explore how our algorithm and approach would change in the presence of tissue, 1.5-mm-thick layers of *ex vivo* bovine tissue were draped over the 3-D model of the uterine arteries, as shown in Fig. 5(b). The handheld optical fiber scanned above this tissue layer, and PA images of vessels 1 to 3 were acquired, first with no tissue covering the vessels and then with increasing layers of the 1.5-mm-thick tissue, up to a thickness of 4.5 mm.

To determine the effect of vessel orientation, images were acquired with the lateral dimension of the probe arranged either orthogonal to the vessel axes, as performed for the experiments described in Sec. 2.3.1 (and shown in the upper right corner of Fig. 3), or parallel to the vessel axes, as shown in Fig. 5(b). Note that these two probe orientations are loosely defined for this second set of experiments, as it is evident from Fig. 5(a) that all vessels are neither perfectly parallel nor orthogonal to the US probe in either orientation.

### 2.4 Fiber Motion

In each trial, the fiber was positioned close to the acoustic window and swept away from it. For the 3-mm vessels, only the proximal and distal boundaries are expected to be visible, due to factors such as constructive interference from subresolution optical absorbers on the target boundary, limited angle view with the linear array, and limited bandwidth of the transducer.^21^^,^^32^ Thus, for these vessels, the goal of image acquisition was to see proximal and distal vessel boundaries rather than entire vessels.

**Fig. 6:**
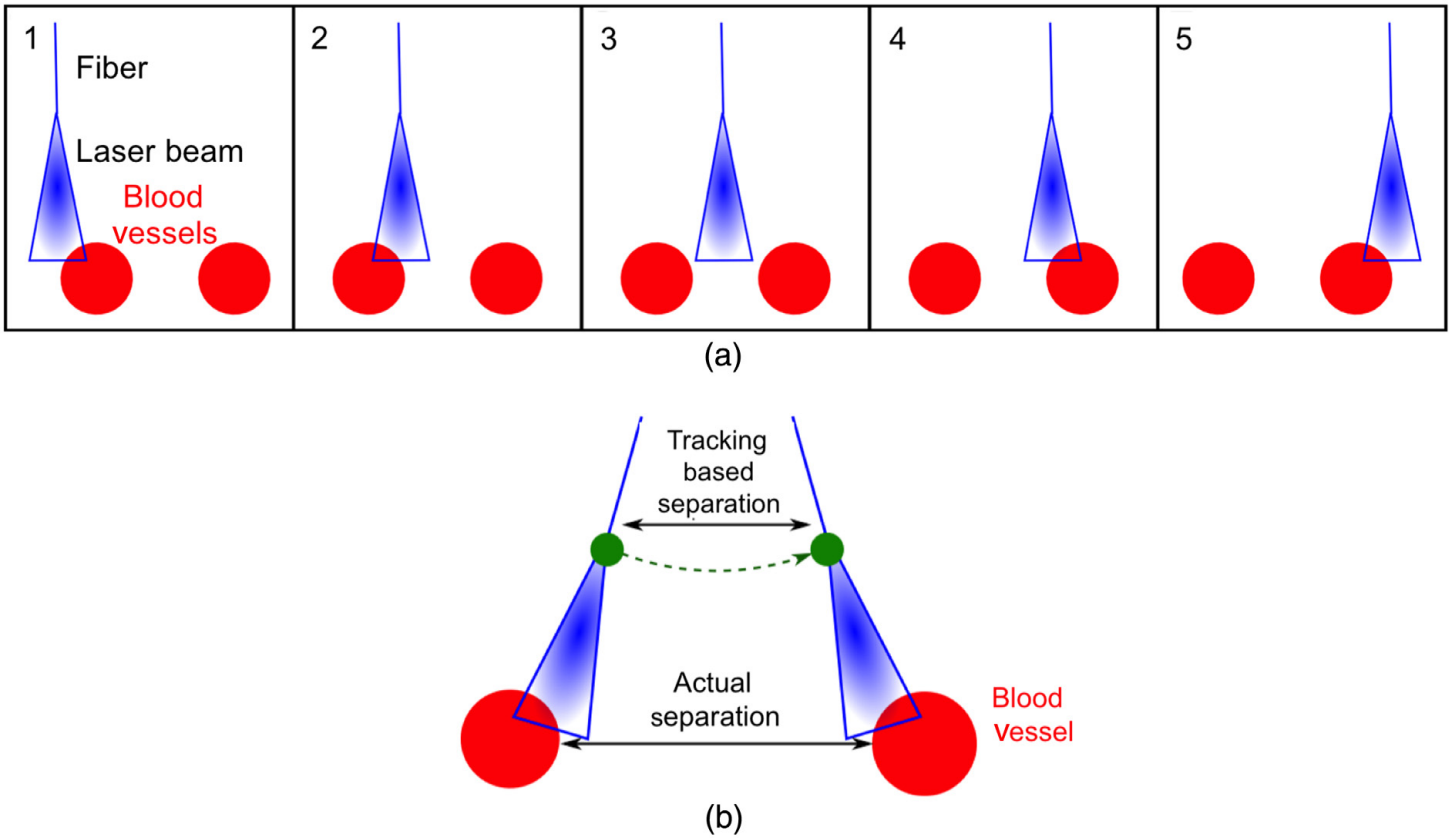
(a) Schematic diagrams of the handheld fiber sweeping from left to right across two blood vessels. (b) Schematic diagram of the fiber sweeping motion with the da Vinci Surgical System. With this system, note that fiber placement directly above the blood vessel is not required to obtain a PA image. The fiber tip and corresponding laser beam profile can be placed along an arc (exaggerated for demonstration purposes) to obtain a PA image of one or more vessel boundaries when the laser beam intersects the blood vessel. The tracking data were acquired to measure vessel separation with the fiber visualizing the inner edges of the two vessels [similar to frames 2 and 4 in Fig. 6(a)], as illustrated by the green dots in Fig. 6(b).

At each vessel boundary, the fiber was held in place while a PA image was acquired. For the teleoperated trials, the corresponding fiber and tool tip location (i.e., tracking data) were available from the da Vinci robot kinematics. A visual representation of the sweeping motion is shown in frames 1 to 5 of Fig. 6(a). For teleoperated trials, the sweeping motion more closely resembles that shown in Fig. 6(b) due to the remote center of motion constraint of the da Vinci surgical system. With this motion, the tracked position of the tool tip does not necessarily correspond to the position of the blood vessel, as illustrated by the two different lengths of the double arrows in Fig. 6(b).

We assume that the fiber will be manipulated above tissue (e.g., within the peritoneal space) and not within the tissue to avoid tissue motion during image acquisition, and more importantly, to avoid imaging-related injury during surgery (e.g., caused by unintended puncturing of tissue with an optical fiber). However, it is also possible to embed the fiber within the tissue if necessary, which would restrict the range of the proposed fiber motion.

### 2.5 Data Analysis

#### 2.5.1 Distance measurements

**Fig. 7:**
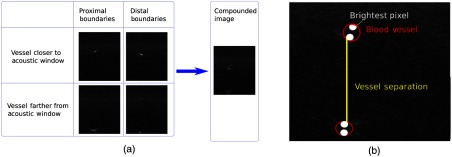
(a) Four PA frames show four different vessel boundaries, representing two blood vessels. These four frames were compounded in a single image. (b) The brightest pixel at each boundary in the compounded image was used to compute vessel separation.

The first set of experiments (performed with both the Ultra100 Nd:YAG laser and the PLD) considered two parallel blood vessel-mimicking targets surrounded by water; four PA images were acquired for each trial. These four images were postprocessed to produce a single compounded image containing all vessel boundaries. We assume that there will be no transducer motion during image acquisition for the compounding step, which is summarized in Fig. 7(a). Once a single compounded image was obtained, the brightest pixel in regions of interest surrounding vessel boundaries was identified. Vessel separation was determined by measuring the distance between the brightest pixels, as illustrated in Fig. 7(b). This distance was measured using images that were processed with both the more conventional amplitude-based delay-and-sum (DAS) beamformer and the innovative coherence-based short-lag spatial coherence (SLSC) beamformer (created with 8% of the receiver aperture), which were both described and compared in our previous publication.^21^ The SLSC beamformer was initially developed for US imaging,^33^ translated to PA imaging,^34^^,^^35^ and demonstrated to improve contrast when performing PA imaging with PLDs,^36^ which are notorious for their low power and poor image quality when utilizing conventional DAS imaging methods.

There are two options for measuring vessel separation distances with the teleoperated system. The first option is based on the da Vinci tracking data, which can be acquired when the vessel boundary is visible in the PA image. This tracking information was collected by the 3-D Slicer software installed on a separate laptop computer (shown in the bottom left of Fig. 3) through an OpenIGTLink connection with the da Vinci research software.^28^ Distance measurements were obtained by recording the two tool tip positions at which the two closest edges of the vessel boundaries were visible in real-time PA images (i.e., one edge per image). This distance measurement can provide information about the extent of available workspace when at least one of the two vessels that were used for the measurement is visible in the PA image. A second option is to determine the location of vessel boundaries directly from PA images (which may be compounded as necessary, as shown in Fig. 7) and use this image-based measurement to determine vessel separation distances. Note that this second option is applied in both manual and teleoperated trials.

When using tracking data from the da Vinci surgical system, which provided an absolute position of the tool tip in 3-D space and, therefore, does not require any postprocessing, vessel separation was calculated as the linear distance between two points in 3-D space as

$$\text{Distance}=\sqrt{{\left(x_{2}-x_{1}\right)}^{2}+{\left(y_{2}-y_{1}\right)}^{2}+{\left(z_{2}-z_{1}\right)}^{2}},$$ (1)

where x, y, and z represent axes in 3-D space, and the subscripts represent the indices of two independent points in space.

For both manual and teleoperated trials, the distance between the fiber and the blood vessel was not controlled, as our primary concern was obtaining a strong PA signal in our image. In addition, due to its remote center of motion constraint, the da Vinci surgical system cannot perform horizontal translations of its instrument shaft, which would be necessary to guarantee that the fiber was the same distance from every vessel boundary in each teleoperated trial. However, fiber placement directly above the blood vessel is not required to obtain a PA image, as demonstrated in our previous publication.^21^ Thus, the fiber tip and corresponding laser beam profile can be placed at multiple locations along the arc as shown in Fig. 6(b), and a PA image of one or both vessel boundaries will be obtained when the laser beam intersects the vessel.

#### 2.5.2 Accuracy measurements

For the first set of experiments, distance measurements from the compounded PA image [shown in Fig. 7(b)] and from the da Vinci tracking data [provided by Eq. (1)] were compared to the ground truth measured with calipers, and the absolute values of these deviations from ground truth were displayed as box-and-whisker plots. Note that we are not using the US image as the ground truth because it is coregistered with the PA image. The horizontal line inside each box displays median error. The upper and lower edges of each box represent the first and third quartiles of the data set. The vertical lines connected to the boxes show the minimum and maximum values in each data set, excluding outliers, which are shown as dots and defined as any value >1.5 times the interquartile range.

The root-mean-square (RMS) error was computed across all trials and vessel separations for each type of data as

$$\mathrm{RMS}\text{ error}=\sqrt{\frac{\overset{\mathrm{NVS}}{\underset{n=1}{\sum }}(\overset{{\mathrm{NVT}}_{n}}{\underset{m=1}{\sum }}D^{2})}{\mathrm{TNVT}}},$$ (2)

where n is the trial number, NVS is the number of vessel separations (4 total), NVT is the number of valid trials in a given vessel separation (at most 10), D is the deviation from ground truth, and TNVT is the total number of valid trials (at most 40). Note that several trials had to be excluded from data analysis calculations due to poor visualization of vessel boundaries in the compounded images.

The mean absolute error (MAE) was computed as

$$\mathrm{MAE}=\frac{\overset{\mathrm{NVS}}{\underset{n=1}{\sum }}(\overset{{\mathrm{NVT}}_{n}}{\underset{m=1}{\sum }}|D|)}{\mathrm{TNVT}}.$$ (3)

#### 2.5.3 Confirmation of accuracy measurements

**Fig. 8:**
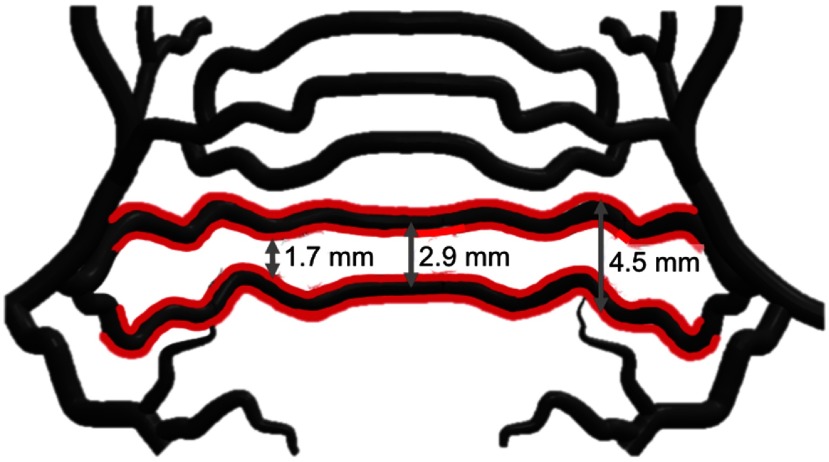
Solid model of vessels 1 and 2 (outlined in red), indicating that there are multiple “ground truth” values.

The second set of experiments with the vascular network was used to confirm accuracy measurements. These experiments were performed with the Phocus Mobile laser. Distance measurements were based solely on the separation between the peak signal amplitudes for vessels 1 and 2. Accuracy measurements were challenged by the presence of multiple “ground truth” values as illustrated in the 3D solid model in Fig. 8, with the vessels of interest outlined in red. We, therefore, compared the vessel separation distances in PA images of these vessels to the three ground truth values shown in Fig. 8, where we identified the minimum separation (measured from inner vessel edges) and maximum separation (measured from outer vessel edges) as 1.7 and 4.5 mm, respectively. The separation between these two vessels at the center of the model (measured from the center of each vessel) is 2.9 mm. While most of our measurements were aimed at the center of the model, this was difficult to confirm when tissue was added, hence we considered multiple ground truth possibilities.

Accuracy for the 3D vessel model were only measured with DAS beamforming and with the probe in the parallel orientation. We expect similar results with SLSC beamforming and with the probe in the orthogonal orientation.

## 3 Results

### 3.1 Compounded DAS and SLSC Images

**Fig. 9:**
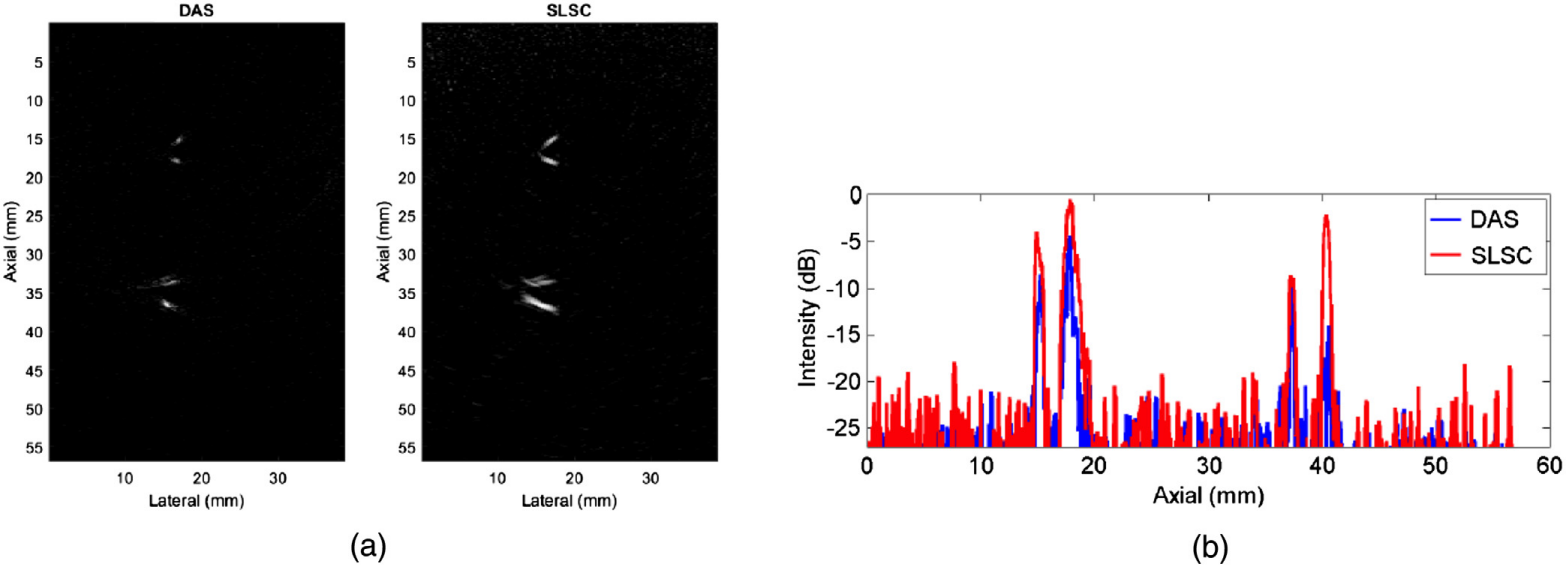
(a) The amplitude-based DAS image shows all four vessel boundaries acquired with the PLD. Visualization of the targets is noticeably enhanced with the coherence-based SLSC image. Both images are displayed with 20-dB dynamic range. (b) Line plots through the same lateral location (16.5 mm) with the four peaks indicating one of the four vessel boundaries. The intensity of the peaks in the SLSC images is generally greater than that of the DAS images, particularly in cases of low DAS peak amplitudes.

An example from a single set of compounded data processed by the DAS and SLSC beamformers is shown in Fig. 9(a). Visualization of the targets is noticeably enhanced with the coherence-based SLSC image when compared to the amplitude-based DAS image. The intensity information as a function of depth for one line, located at lateral position 16.5 mm, is shown in Fig. 9(b). The four peaks in each line plot indicate one of the four vessel boundaries. Note that the intensity of the signal peaks in the SLSC image is generally greater than that of the DAS images, particularly in cases of low DAS peak amplitudes. The noise floor appears to be higher with SLSC beamforming because the associated correlation calculations tend to fluctuate around a minimum value (which is amplified with compounding) whereas in DAS, the mean of the noise floor is generally zero.

### 3.2 Accuracy Results

**Fig. 10:**
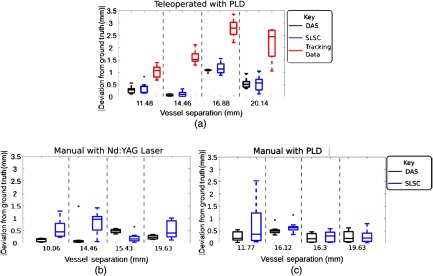
Four different vessel separations were tested. (a) The deviation from known vessel separations was measured using tracking data from the da Vinci and PA images acquired with the PLD. Similar results were obtained when manually sweeping an optical fiber coupled to the (b) Nd:YAG laser and (c) PLD.

Figure 10(a) shows results from experiments with the optical fiber coupled to the PLD and attached to the da Vinci tool tip. For the separation measurements derived from the tracking data of the teleoperated trials, there is an apparent increase in the deviation from ground truth values as the vessel separation distance increases, particularly for separation distances ranging from 11.48 to 16.88 mm for the teleoperated trials.

Figure 10(b) shows results from manually sweeping the optical fiber when coupled to the Nd:YAG laser, while Fig. 10(c) shows results when manually sweeping the optical fiber coupled to the PLD. These two results, in tandem with the image-based results in Fig. 10(a), demonstrate that there is no definitive trend in the magnitude of error as vessel separation increases. Each DAS and SLSC PA image generally provides more accurate and precise estimation of vessel separation when compared to measurements obtained with the da Vinci tracking results.

For the data analysis method chosen here (i.e., evaluations based on the brightest pixels), DAS images generally provided higher accuracy (determined by closeness of the median to 0 mm) and precision (determined by range, excluding outliers) relative to SLSC images, particularly when imaging with the Nd:YAG laser [Fig. 10(b)]. However, SLSC images resulted in 45% fewer trials excluded from data analysis calculations due to poor visualization of vessel boundaries in the compounded images, as summarized in Table 1, which lists the number of trials excluded for each vessel separation tested. Note that experiments with the Nd:YAG laser also resulted in fewer excluded trials relative to experiments with the PLD, likely due to the higher laser energy (0.75 mJ versus 6.6  μJ), which increases signal-to-noise ratios.

**Table 1:** Summary of trials excluded from data analysis calculations due to poor visualization of one or more vessel boundaries in the compounded PA image.

	Separation distance (mm)	Number of excluded trials	
		DAS	SLSC
Teleoperated sweep with PLD	11.48	3	3
	14.46	1	0
	16.88	0	0
	20.14	0	0
Manual sweep with Nd:YAG laser	10.06	0	0
	14.46	1	1
	15.43	0	0
	19.36	0	0
Manual sweep with PLD	11.77	3	2
	16.12	0	0
	16.30	1	0
	19.63	2	0
Total number of excluded trials	—	11	6

**Fig. 11:**
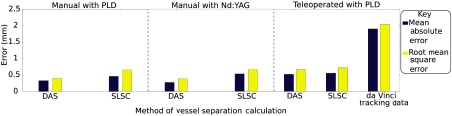
Summary of MAE and RMS error for the three experiments.

When comparing RMS error, the SLSC beamformer provides a 64.78% improvement in accuracy compared to the tracking data, while the DAS beamformer provides a 67.56% improvement in accuracy compared to the tracking data, as demonstrated in Fig. 11. The RMS error for the tracking data is 2.04 mm. Similar improvements in accuracy (70.74% and 72.47%, respectively) were observed when considering the MAE, which was 1.90 mm for the tracking data, as shown in Fig. 11.

### 3.3 Effects of Probe Orientation and Tissue Thickness

**Fig. 12:**
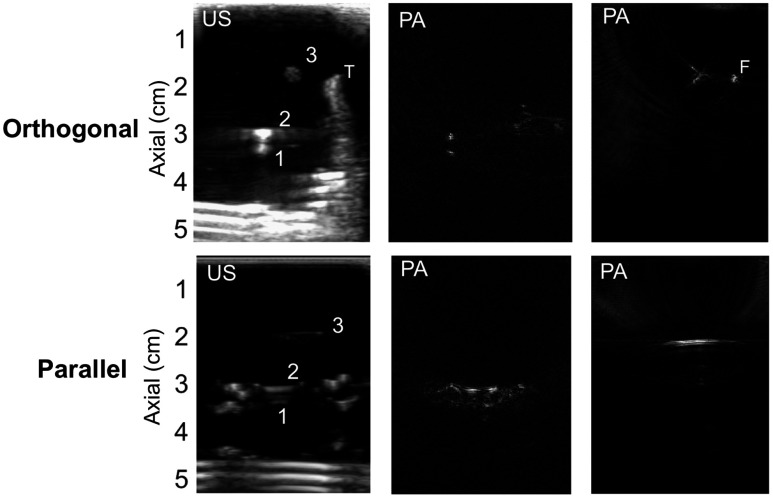
Sample US and PA images demonstrating vessels 1 to 3 in orthogonal and parallel orientations. The tissue thickness for these US and PA images is 1.5 mm. All PA images were acquired with ∼1.58-mJ energy at the tip of the fiber (T = tissue and F = signal from fiber).

The vascular network and *ex vivo* tissue were added to the custom phantom container in order to provide an assessment of performance in a more realistic environment. Sample images from one layer of tissue (1.5-mm thickness) are shown in Fig. 12. When the probe was in an orthogonal orientation, the vessels and tissue boundaries were visualized in the US image. When the fiber is in place, it can be visualized in the PA images in this orientation (noted as F in Fig. 12).

The parallel probe orientation provides additional information to help distinguish one large vessel (e.g., vessel 3, which shows proximal and distal boundaries in PA images with an orthogonal probe orientation) from two small vessels (e.g., vessels 1 and 2). Based on these two views, it is apparent that the top two signals in the orthogonal orientation arise from a single straight vessel, and the bottom two signals in the orthogonal orientation are associated with two smaller vessels with curved geometries. This result indicates that it will be possible to differentiate one big vessel with two boundaries from two small vessels during surgery (when these vessels are hidden beneath a tissue surface) if the US probe can be rotated 90 deg to align with the suspected vessel axis, as shown in Fig. 12. This distinction can also be made from images in the orthogonal orientation if the subtle difference between the two types of vessel boundaries (i.e., point-like for smaller vessels versus a subtle linear stroke for larger vessels) are translatable to and distinguishable in an *in vivo* environment.

**Fig. 13:**
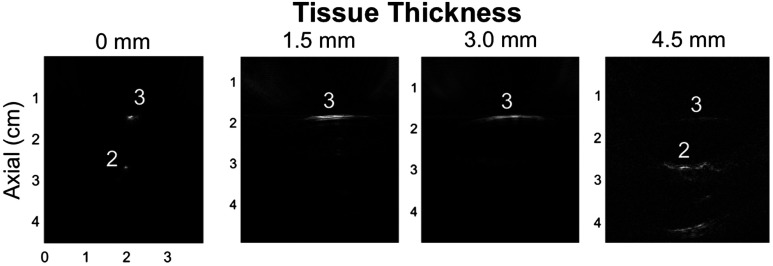
Sample PA images acquired with the probe in the parallel orientation for a range of tissue thicknesses. The tissue thickness is listed above each image. Notice that the PA signal is more evenly distributed across the vessels as the tissue thickness increases. All images were acquired with ∼0.75-mJ energy at the tip of the fiber.

With the probe in the parallel orientation, we can also appreciate how the signal changes as a function of tissue thickness, as shown in Fig. 13. When there is no tissue present, the signal looks similar to the signals observed in the orthogonal orientation. However, the presence of tissue diffuses the light, and the length of the vessel is more apparent when tissue is added. Because of this diffusion and the smaller separation distances in this second set of experiments, compounding multiple images was not applied when tissue was present.

**Fig. 14:**
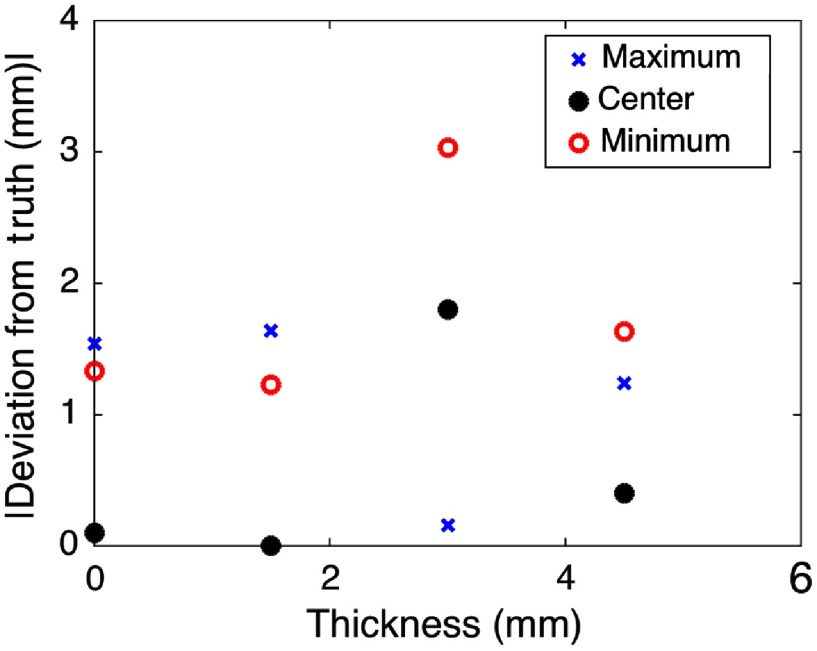
The distance between vessels 1 and 2 was compared to the three ground truth values shown in Fig. 8 (maximum, center, and minimum) and plotted as a function of tissue thickness. These results show that at least one value for each thickness is within the accuracy achieved with the best-case scenario.

Despite the significant differences in vessel appearance in the presence of tissue, we used our proposed method to extract the image-based positions of the highest-amplitude signals for each boundary observed. We only used DAS beamforming for these separation measurements because the results in Sec. 3.2 show that DAS performs slightly better than SLSC when using an algorithm that searches for the highest pixel intensities within a region of interest. The measured distance between vessels 1 and 2 (obtained with the probe in the parallel orientation) was compared to the three ground truth values shown in Fig. 8. This comparison is plotted as a function of tissue thickness in Fig. 14, and results show that at least one or more of the values are within the maximum, RMS, and MAE accuracies achieved in the best-case scenario (compare Fig. 14 with Figs. 10 and 11). Specifically, the accuracy in Fig. 14 ranges from 0 to 3 mm.

## 4 Discussion

Our approach to surgical guidance has the potential to rely purely on a real-time PA imaging system that determines the separation distance among critical anatomical features (e.g., blood vessels or nerves). This separation is most useful when both the features and the tool tip are visible in the same image, and we previously demonstrated that this type of simultaneous visualization is possible with a specialized light delivery system.^22^ Although we demonstrated that our real-time PA image guidance approach may be interfaced with a da Vinci Surgical System, this approach is translatable to any robotic surgical system.

While we demonstrated feasibility with an external US probe with 3- to 8-MHz bandwidth, higher US frequencies may also be used, particularly if the probe is placed at the site of surgery. However, we consider demonstration with an external, lower-frequency US probe to be a more significant accomplishment because conventional PA tomography and PA microscopy systems have consistently utilized high-frequency US transducers, which can be extended to demonstrate the feasibility of visualizing multiple vessels at high resolution (therefore determining their separation). Our work, however, is the first to demonstrate that vessel visualization, and an automated target separation measurement is possible for surgical guidance when the light source is separated from a lower-frequency, larger-aperture US transducer that will be placed externally and, therefore, will require deep acoustic penetration depths. As a result, success with the lower frequency, external probe is the primary focus of the method shown in this paper. This probe could be a conventional linear, curvilinear, or phased array US probe. It could also be a transvaginal US probe for imaging the uterine arteries during gynecological surgeries.

Image-based vessel separation calculations for the first set of experiments were performed with DAS and SLSC images. DAS images generally provided greater accuracy than SLSC images because the data analysis was biased toward amplitude information, while SLSC images are known to be independent of signal amplitude. However, the SLSC images increased target visibility, resulting in fewer trials excluded from the final calculations, representing a trade-off between using SLSC images for image visualization and DAS images for amplitude-based data analyses. For real-time navigation, SLSC imaging would likely be more beneficial due to the higher contrast images. Results indicate that PA image guidance provides submillimeter MAE and RMS errors when determining the separation of critical landmarks, thus providing the potential to maximize surgical workspace while minimizing damage. Results additionally indicate that PA image-based measurements are more reliable than measurements derived from the image-based tracking data obtained from the da Vinci kinematics, as shown in Figs. 10(a) and 11. Although the image-based measurements require additional postprocessing when compared to measurements obtained with tracking data, the increase in accuracy substantiates the additional time and computational expense required to perform image-based measurements. With the addition of tissue (for the second set of experiments), it is evident that this compounding step, postprocessing, and separation calculations may not always be needed, particularly if nearby vessels and the tool tip (or optical fiber) are simultaneously visualized with only one fiber position (as shown in Figs. 12 and 13).

In general, the separation measurements investigated in this paper are important to provide the surgeon with information about safety zones for operation, with the required accuracy depending on the particular surgical task (e.g., when differentiating internal carotid arteries during transsphenoidal surgeries, which have a reported separation of 4 to 18 mm,^31^ an accuracy <1  mm seems to be sufficient). When the fiber is manipulated manually, this information is only available in the image coordinate system, therefore, the surgeon can primarily use it for assurance regarding the extent of available workspace. This information may also be used to determine tool-to-vessel distances if the tool tip is simultaneously present in the images. If, however, the fiber is attached to a robot or tracked by a navigation system, it is possible to provide vessel locations in robot or navigation system coordinates by registering the PA imaging system to the robot (or navigation system) so that features located in the PA image can be transformed to robot coordinates. This approach requires calibration of the probe to the robot coordinate system and, therefore, introduces additional sources of error.^24^ An alternative approach is to use the PA image as a real-time sensor signal that indicates whether or not a critical structure is in the laser path, similar to the robotic approach in this paper. Because the optical fiber delivering the laser light is fixed to the robot instrument, it would be possible to accurately calibrate the fiber with respect to the robot and locate the centerline between two vessels with respect to the robot coordinate system.^25^ Another option is to overlay a cross section of the tool model on the real-time PA images displayed in the robot coordinate system, which is particularly advantageous when the surgical tool tip is unable to be visualized in the PA image.

There are four key clinical implications when integrating the proposed PA image guidance system into existing surgical workflows. First, the proposed sweeping motion and associated separation measurements can provide the surgeon with assurance regarding the extent of available workspace when the optical fiber is either manually operated or teleoperated (assuming that there is enough room to visualize two critical structures in one fiber position or in one sweep of the fiber). Second, the PA system can be developed to provide indications about where to operate (or where not to operate), as the presence of a vessel boundary would indicate that the surgeon should update the plan for making incisions or change the direction of drilling, and the absence of a vessel boundary would indicate that the surgeon is operating in a safety zone (assuming that the US probe is correctly positioned to visualize the region of interest). An additional assumption when bone is present is that the bone is thin enough for PA imaging.^22^ Third, with the integration of a robotic system, the surgeon would have quantitative information about the center of the safety zone in the robot coordinate system.^25^ This integration is advantageous because the surgeon can obtain information about the vessel locations with respect to the robot-held surgical tool (which would have one or more optical fibers attached). Similar information may be obtained with the image-based coordinate system if features such as the tool tip are also visible in the PA image.^22^

## 5 Conclusion

PA image guidance is a feasible alternative or adjunct to existing image guidance modalities, such as US, CT, and MRI. The ability to clearly visualize hidden vessels and nerves with real-time PA imaging can reduce the incidence of morbidity and mortality in a wide range of minimally invasive surgeries, including endonasal transsphenoidal surgery, mastoidectomies, and gynecological surgeries. In telerobotic operations, the addition of real-time PA image guidance improves the accuracy of separation measurements when compared to the tool position tracking information provided by the robot kinematics. Results are promising for real-time path planning in both robotic and nonrobotic interventional PA applications.
